# Failure after proximal humeral fracture osteosynthesis: a one year analysis of hospital-related healthcare cost

**DOI:** 10.1007/s00264-020-04577-y

**Published:** 2020-04-28

**Authors:** Jan Dauwe, Gregory Walters, Lukas A. Holzer, Kris Vanhaecht, Stefaan Nijs

**Affiliations:** 1 AO Research Institute, Davos, Switzerland; 2 Department of Orthopedic Surgery, University Hospitals Leuven, Leuven, Belgium; 3 Department of Trauma Surgery, University Hospitals Leuven, Leuven, Belgium; 4 Department of Orthopaedic Surgery, AUVA Trauma Center Klagenfurt, Waidmannsdorferstraße 35, 9020 Klagenfurt am Wörthersee, Austria; 5 Department of Orthopaedics and Trauma, Medical University of Graz, Graz, Austria; 6 Institute for Healthcare Policy, University of Leuven, Leuven, Belgium

**Keywords:** Healthcare cost, Cost analysis, Proximal humeral fracture, Osteosynthesis, Failure

## Abstract

**Purpose:**

The issue of rising healthcare costs and limited resources is a topic of worldwide discussion over the last several decades. We hypothesized that failure of proximal humeral fracture osteosynthesis is presumed to be an important determinant in healthcare resources and related costs. The aim of this study was to calculate the total hospital-related healthcare cost of proximal humeral fracture osteosynthesis over one  year focusing on failure.

**Methods:**

A total of 121 patients with a proximal humeral fracture treated by angular stable osteosynthesis were included in this retrospective study. All hospital-related healthcare costs were investigated. Five main hospital-related cost categories were defined: hospitalization cost, honoraria, day care admission, materials, and pharmaceuticals.

**Results:**

A total healthcare cost of € 1,139,448 was calculated for the whole patient group. Twelve patients needed revision surgery due to complications or fixation-related failure. This failure rate alone costed € 190,809 of the healthcare resources. In other words, failure after proximal humeral fracture osteosynthesis costed 17% of the total healthcare expenditure inone year.

**Conclusion:**

This study demonstrates that a high amount of hospital-related healthcare resources is spent because of failure after proximal humeral fracture osteosynthesis. Further research is necessary and should investigate on how to prevent failure. This is not only in the patient’s interest, but it is also of great importance for maintaining a healthy healthcare system.

## Introduction

The issue of rising healthcare costs and limited resources is a topic of worldwide discussion over the last several decades. Reports and studies concerning healthcare expenditure have been published suggesting that possible cost cutting measures will be mandatory in the near future. According to the data of 2018, Belgium is number nine on the list of OECD (Organization for Economic Co-Operation and Development) countries spending 10.4% of their GDP (gross domestic product) on healthcare expenses [1]. Cancer and cardiovascular diseases remain the leading causes of mortality in Belgium. However, musculoskeletal problems (e.g., proximal humeral fractures) can have serious consequences on health-related quality of life. Proximal humeral fractures (PHF) currently account for approximately 5% of all fractures in adults and up to 10% in the elderly [2, 3]. The amount of proximal humeral fractures will continue to increase since the elderly population is growing, resulting in an even higher healthcare resource utilization. Most of the PFH can be treated conservatively; however, in displaced fractures, angular stable osteosynthesis is recommended. The results of surgical treatment have been improving due to advancements in operative procedures and implant design. Nevertheless, failure rates after osteosynthesis of proximal humeral fractures are still high, ranging up to 35% [4–11]. In the present study, we hypothesized that the failure of proximal humeral fracture osteosynthesis is presumed to be an important determinant in healthcare resources and related costs. The aim of this study was to calculate the total hospital-related healthcare cost of PHF osteosynthesis over one year with a focus on failure.

## Materials and methods

### Patients

After approval of the ethical committee of the University Hospitals of Leuven, a total of 121 patients with the diagnosis of a proximal humeral fracture were included in the current retrospective study. Clinical data were safely obtained from the database KWS (Klinisch WerkStation). Only indications for angular stable osteosynthesis were included. All patients were treated at the Department of Trauma Surgery between January 2017 and January 2018. Patients presenting with additional injuries next to a sole proximal humeral fracture were excluded. In the present analysis, all hospital-related healthcare costs were included.

### Surgical implants

Three types of angular stable devices were used for treatment of proximal humeral fractures. The Philos plate® (AO Synthes GmbH, Oberdorf, Switzerland) and ALPS plate® (Zimmer-Biomet, Warsaw, USA) were used for angular stable plate osteosynthesis.

The Multiloc Proximal Humeral Nail® (AO Synthes GmbH, Oberdorf, Switzerland) was the device used for angular stable intramedullary nailing.

### Study variables

Ten variables were recorded and studied. The clinical variables were grouped as patient characteristics (gender, age, ASA [American Society of Anesthesiologists] score, AO/OTA [Arbeitsgemeinschaft für Osteosynthesefragen/Orthopedic Trauma Association] fracture type, type of definite treatment, failure rate, cause of failure, and two other variables (total LOS [length of stay], total LOS per patient)).

The ASA score is commonly used to assess patient comorbidity. Based on computed tomography (CT), all fractures were classified according to the AO/OTA classification. The type of definite surgery was categorized as plate-screw osteosynthesis or intramedullary nail fixation. Failure was defined as a post-operative complication which required re-operation. Causes of failure were classified as non-union, infection-, or fixation-related causes (e.g., implant loosening, screw pull-out/penetration, impingement, cuff tear). Note that not all non-failure cases were completely successful. Non-failure was defined as a result after osteosynthesis which met the needs of the individual patient depending on his/her daily life activities. Finally, LOS was defined as the total number of consecutive hospital admission days during the stay for the definite treatment.

### Cost categories

Five main hospital-related cost categories were defined: hospitalization (cost of daily patient care), honoraria, day care admission, materials, and pharmaceuticals. These cost categories are shown in Table 2. The honoraria category mainly consists of fees related to medical activities (i.e., surgery, consults, and imaging), based on a fee-for-service principle. In Belgium’s healthcare system, honoraria are independent from the rank of the surgeon as activities are billed under the attending physician. Material-related costs involve the costs of the actual implants and other materials used peri-operatively. Pharmaceutical costs are the costs for received drugs and blood products.

The calculated costs in this paper are limited to the hospital-related costs covered by the Belgian healthcare financing system. Furthermore, all costs investigated in this study are defined as the total reimbursements paid to the hospital by any party involved in financing the care for a specific patient either directly or indirectly.

## Results

Table 1 shows a detailed overview of the characteristics of all included patients. This group consisted of 121 patients with an average age of 65 years. Sixty-seven percent were female and 33% male. As mentioned earlier, all proximal humeral fractures were treated with angular stable osteosynthesis. There were no open proximal humeral fractures. In almost 60% of the cases, fracture treatment was performed with a locking plate-screw osteosynthesis. In the rest of the cases, an angular stable intramedullary nail was preferred. Cement augmentation for extra stability was not used; however, three cases were treated with allograft. In two failed cases, fibular allograft was used whereas in one non-failure case, femoral head allograft was chosen. The AO/OTA fracture type 11.C was the most common fracture accounting for 45% of all PHF in our analysis.

**Table 1:** Patient characteristics

Gender	
Male	40 (33.1%)
Female	81 (66.9%)
Age	65 years
ASA score	
ASA 1	20 (16.5%)
ASA 2	57 (47.1%)
ASA 3	39 (32.2%)
ASA 4	5 (4.2%)
AO/OTA classification	
11.A1	7 (5.1%)
11.A2	33 (27.3%)
11.A3	5 (4.2%)
11.B1	22 (18.2%)
11.C1	29 (24.0%)
11.C3	25 (20.6%)
Prophylactic antibiotic therapy	121 (100%)
Type of osteosynthesis	
Locking plate	71 (58.7%)
Intramedullary nail	50 (41.3%)
Allograft use	3 (2.5%)
Number of failures	12 (9.9%)
Cause of failure	
Infection	4 (3.3%)
Non-union	2 (1.7%)
Others (fixation related)	6 (5.0%)
Total LOS	974.88 days
Total LOS per patient	8.1 days

Categorical variables are presented as numbers and percentages, continuous variables as average. *ASA score* American Society of Anesthesiologists score, *AO/OTA* Arbeitsgemeinschaft für Osteosynthesefragen/Orthopedic Trauma Association, *LOS* length of stay

Twelve patients needed revision surgery due to complications or fixation-related failure leading to a failure rate of almost 10%.

The total length of stay (LOS) amounted 975 days which equates to circa eight days per patient.

A total healthcare cost of € 1,139,448 was calculated for our patient cohort in one year. This is the equivalent of € 9417 average per patient. The total expenditure includes hospitalization cost, day care admission, material, honoraria, and pharmaceutical products. Hospitalization cost accounted for almost 55% (Table 2).

**Table 2:** Healthcare costs per category for 121 patients over 1 year

Category	Per patient	Total	Relative share
Honoraria	€ 2074	€ 251,012	22.0%
Day care admission	€ 151	€18,322	1.6%
Materials	€ 1394	€ 168,682	14.8%
Hospitalization	€ 5147	€ 622,773	54.7%
Pharmaceuticals	€ 650	€ 78,659	6.9%
Total	€ 9417	€ 1,139,448	100%

Table 3 focuses on failure after proximal humeral fracture osteosynthesis. Twelve patients needed revision surgery because of failure and eight underwent multiple operations in the year from January 2017 to January 2018. Taking the primary and revision cases into account performed during our one year search period, € 34,150 was spent after initial plate fixation whereas € 63,198 was spent after primary nail fixation. A total amount of € 190,809 was spent because of complications and fixation-related failure. In other words, failure after proximal humeral fracture osteosynthesis costed 17% of the total healthcare expenditure in one year.

**Table 3:** Healthcare cost of 12 patients with failure after osteosynthesis. Operations are presented as rows per case

	Primary/revision surgery: operation type	Honoraria	Day care admission	Materials	Hospitalization	Pharmaceuticals	Length of stay (days)	Total cost per case	Total cost
Case 1	Primary: Philos plate	€ 1117	€ 69	€ 61	€ 2011	€ 187	29	€ 15,820	€ 190,809
	Revision: implant removal + Latarjet	€ 1960	€ 157	€ 1670	€ 7375	€ 1213	11.3		
Case 2	Primary: nail	€ 1807	€ 125	€ 1128	€ 3352	€ 460	5.2	€ 15,170	
	Revision: implant removal + reversed shoulder prosthesis	€ 1819	€ 125	€ 2329	€ 3352	€ 673	5.2		
Case 3	Primary: nail	€ 1464	€ 147	€ 1333	€ 4023	€ 984	5.9	€ 28,202	
	Revision: irrigation and debridement	€ 790	€ 163	€ 47	€ 2011	€ 199	2.3		
	Revision: implant removal + cement spacer	€ 1789	€ 189	€ 70	€ 14,080	€ 913	20.8		
Case 4	Primary: nail	€ 1404	€ 134	€ 1021	€ 4023	€ 1266	6.1	€ 9445	
	Revision: screw extraction + MON	€ 487	€ 90	€ 0	€ 670	€ 350	1.4		
Case 5	Primary: Philos plate	€ 1033	€ 92	€ 199	€ 2682	€ 213	3.9	€ 9177	
	Revision: cuff repair	€ 1598	€ 102	€ 927	€ 2011	€ 320	3.1		
Case 6	Primary: nail	€ 1838	€ 104	€ 1137	€ 3352	€ 360	4.7	€ 10,381	
	Revision: implant removal	€ 591	€ 88	€ 0	€ 2682	€ 229	4.0		
Case 7	Revision: Philos plate (refracture)	€ 1713	€ 111	€ 1286	€ 3352	€ 412	4.9	€ 26,129	
	Revision: irrigation and debridement	€ 2429	€ 130	€ 1321	€ 14,750	€ 625	21.7		
Case 8	Revision: removal nail + Philos plate + fibula graft	€ 2010	€ 153	€ 1189	€ 8716	€ 1797	13.0	€ 13,865	
Case 9	Primary: Philos plate	€ 954	€ 91	€ 682	€ 1341	€ 273	2.1	€ 9153	
	Revision: new Philos plate + cuff repair	€ 1432	€ 94	€ 961	€ 2682	€ 643	3.9		
Case 10	Revision: Alps plate + fibula graft	€ 1794	€ 118	€ 1218	€ 9386	€ 1559	13.9	€ 14,075	
Case 11	Revision: screw extraction (nail) + cuff repair + MON	€ 1164	€ 93	€ 998	€ 2011	€ 364	2.9	€ 4630	
Case 12	Revision: implant removal (cement nail) + 2nd time reversed shoulder arthroplasty	€ 3513	€ 277	€ 2152	€ 27,489	€ 1331	41.1	€ 34,762	

*MON* manipulation under narcosis

## Discussion

Proximal humeral fractures (PHF) are the most common type of humeral fractures in adults [12]. Angular stable osteosynthesis is currently the gold standard in joint-preserving surgery [10]. The goal is to stabilize the fracture, aid better union, and reduce pain during the healing process. However, open reduction and internal fixation of PHF remains a challenging task in trauma surgery. As mentioned above, failure rates range up to 35% reported in the literature [4–11]. One of the contributing factors to the high healthcare expenditure are these musculoskeletal complications or failure after surgery. Therefore, researchers find an increasing interest in this extended and global topic since healthcare resources are becoming more limited. The aim of the present study was to investigate the impact of failure after proximal humeral fracture osteosynthesis to our healthcare resources.

In this exploratory analysis, we found that the hospitalization cost is the most important factor in total healthcare cost of proximal humeral fracture osteosynthesis. The relative share of the latter cost category is calculated at 55% of the total healthcare costs. A similar finding was found by Smeets et al. [13] in their analysis on healthcare costs and fibular plating for AO/OTA type 44-B fractures. Hospitalization costs accounted for circa half of the total healthcare expenses followed by honoraria and pharmaceutical products. Another analytic study demonstrated a relative share for hospitalization costs of 62% [14]. This hospitalization cost weighs the most in the total hospital-related healthcare expenditure because of the expensive days spending in the hospital (defined as length of stay).

In comparison with other studies where the cost of infection in tibia fracture fixation was investigated [14, 15], we estimated the hospital-related cost for failure meaning every post=operative complication that required revision surgery. Four cases of infection were included in our analysis.

Based on this data, a simplified cost-effectiveness analysis could be performed comparing the relative costs with the outcome after the intervention (or effect of the investment). Although data are lacking, it is interesting to discuss an intuitive (qualitative) cost-effectiveness analysis. The data necessary for a cost-effectiveness analysis are presented in Table 3. For example, in case 4, the patient is relieved from pain six weeks after the revision operation that costed circa € 9500. Compared to patient 10, the same pain outcome was found; however, this treatment costed € 5000 more in total hospital-related costs. Patient 11 can be considered as the most cost-effective case regarding shoulder function: a total cost of € 4630 (lowest cost of all the failure cases) was calculated for a gain from zero to a good shoulder function. This patient is followed by patient 5 who achieved a full range of motion after one year (€ 9177) and patient 9 attaining an almost full function after 6 months (€ 9153). Furthermore, according to our data, the infection cases can be considered as the least cost-effective ones since their total costs are unquestionably higher (mostly due to the hospitalization cost as discussed above).

Note that it is highly challenging to compare international healthcare systems and generalize our data towards other countries. Belgium has a specific care financing system. Hospitals are mostly financed through the Ministry of Health and the healthcare insurance system (75%). Only a minimal part is paid by patient co-payments [16, 17]. Although Belgium currently has a more cost-based financing system, it is moving towards a prospective system where healthcare expenditure awareness plays a leading role [13].

There are several limitations of this analysis requiring some explanation. Our patient cohort consists of 121 patients who were investigated retrospectively. This is a rather small amount since all patients with more than a sole proximal humeral fracture were excluded. Nevertheless, this is necessary because our results (such as total LOS, number of operations) would be compromised otherwise. This study is an exploratory analysis meaning that the goal was not to compare treatment strategies in order to find the most cost-effective treatment option. Our aim was to calculate the total hospital-related cost over one year with a focus on failure after PHF osteosynthesis.

However, to the best of our knowledge, no such study was found in medical literature assessing all hospital-related costs in proximal humeral fracture osteosynthesis. Moreover, increasing interest in the operation of healthcare systems and the rising awareness of healthcare expenditure should be encouraged. Further research is mandatory in the field of healthcare utilization and related costs. The present study specifically demonstrates that a high amount of hospital-related healthcare resources (€ 190,809) is spent because of failure after proximal humeral fracture osteosynthesis. It is not only in the patient’s interest, but it is also of great importance for socio-economic reasons that more research is conducted to prevent failure.
